# HDAC Inhibitors: Dissecting Mechanisms of Action to Counter Tumor Heterogeneity

**DOI:** 10.3390/cancers13143575

**Published:** 2021-07-16

**Authors:** Dimitris Karagiannis, Theodoros Rampias

**Affiliations:** 1 Department of Genetics and Development, Columbia University Medical Center, New York, NY 10032, USA; 2 Biomedical Research Foundation of the Academy of Athens, 11527 Athens, Greece

**Keywords:** cancer, tumor heterogeneity, epigenetic drugs, HDAC inhibitors

## Abstract

**Simple Summary:**

Tumor heterogeneity promotes the development of drug resistance in cancer. HDAC inhibitors modulate several processes that contribute to intra-tumoral heterogeneity. With careful consideration of the underlying biology, HDAC inhibitors can be utilized to improve therapeutic efficacy.

**Abstract:**

Intra-tumoral heterogeneity presents a major obstacle to cancer therapeutics, including conventional chemotherapy, immunotherapy, and targeted therapies. Stochastic events such as mutations, chromosomal aberrations, and epigenetic dysregulation, as well as micro-environmental selection pressures related to nutrient and oxygen availability, immune infiltration, and immunoediting processes can drive immense phenotypic variability in tumor cells. Here, we discuss how histone deacetylase inhibitors, a prominent class of epigenetic drugs, can be leveraged to counter tumor heterogeneity. We examine their effects on cellular processes that contribute to heterogeneity and provide insights on their mechanisms of action that could assist in the development of future therapeutic approaches.

## 1. Introduction

### 1.1. HDAC Inhibitors in Cancer Therapy

Histone deacetylase inhibitors (HDACi) were the first class of epigenetic drugs to be approved for cancer therapy. Currently, four HDACi, vorinostat, belinostat, Panobinostat, and romidepsin, are approved by the FDA for cancer treatment, and several others are under investigation in the clinic (Table 1). Vorinostat is approved for use in refractory cutaneous T-cell lymphoma (CTCL) [1], belinostat for refractory peripheral T-cell lymphoma (PTCL) [2], and romidepsin for CTCL and PTCL patients that have received one prior therapy [3,4]. Panobinostat is approved as a third-line treatment in multiple myeloma (MM) in conjunction with bortezomib and dexamethasone. Since their discovery, a huge effort has been underway to expand their use in cancer therapy. Despite their initial success as monotherapy in these hematological cancers, the current clinical investigations using HDACi have been reshaped to drug combination strategies (reviewed here [5,6,7]). So far, ongoing and completed studies have examined combinations of HDACi with chemotherapy, DNA methylation inhibitors, tyrosine kinase inhibitors, estrogen inhibitors, immunotherapy, etc. Prominent phase III clinical trials include studies on the treatment of hormone receptor-positive and HER2-negative breast carcinoma with Class I-selective HDACi entinostat and tucidinostat in combination with aromatase inhibitor exemestane (NCT02482753 [8], NCT02115282 [9]), and studies examining the activity of vorinostat and VPA in pediatric high-grade glioma in combination with temozolomide (NCT03243461, NCT01236560). In this review we give an overview of the current knowledge on the mechanisms of action of HDAC inhibitors and propose ways they could be leveraged in future clinical applications, in the context of tumor heterogeneity and therapy resistance.

**Figure 1:**
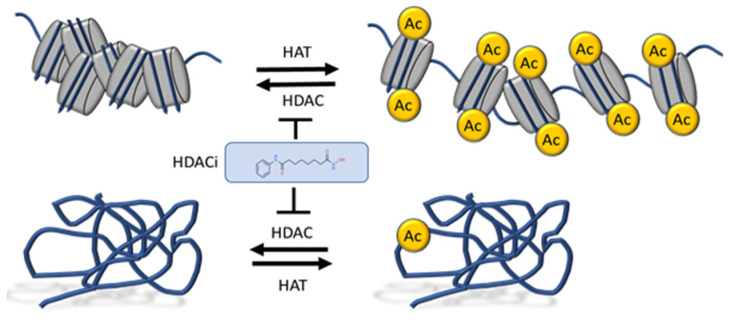
A schematic depicting how HDAC inhibitors modulate histone acetylation in relation to chromatin structure and acetylation of non-histone proteins. HAT: Histone Acetyltransferase; HDAC: Histone deacetylase; HDACi: Histone deacetylase inhibitors.

**Figure 2:**
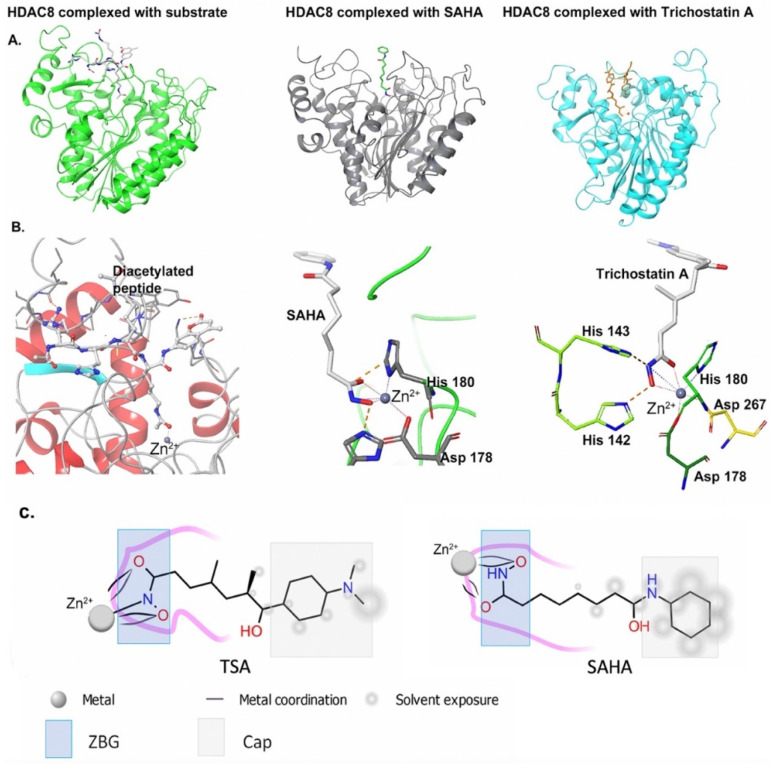
HDAC inhibitor coordination to the active site metal Zn^2+^ ion. (**A**) HDAC8 structures showing the binding sites of substrate (**left**, PDB: 2V5W), SAHA (**center**, PDB: 1T69), and trichostatin A (**right**, PDB: 1T64) (colored small molecules). (**B**) Stereoviews showing substrate carbonyl (**left**) and HDAC inhibitors (**center** and **right**) coordination to the catalytic Zn^2+^. (**C**) Zinc binding group and cap group in SAHA and trichostatin A inhibitors. TSA: Trichostatin A; SAHA: Suberoylanilide Hydroxamic Acid.

Histone acetylation is an epigenetic modification important for gene expression, genome maintenance, and DNA replication. Several lysine residues in histones 3 (e.g., H3K27 and H3K9) and 4 (e.g., H4K16, H4K12) are acetylated, and are generally associated with a permissive chromatin state. These modifications are deposited by histone acetyltransferase complexes, such as CBP/p300 and GCN5, and removed by histone deacetylases, which include HDAC and Sirtuin (SIRT) enzymes (Figure 1) (reviewed in [19]). Class I HDACs include HDAC 1, 2, 3, and 8, which are ubiquitously expressed, localize primarily in the nucleus, and are components of multiple repressor complexes [20]. Class II HDACs display a tissue-specific pattern of expression, localize in both the nucleus and the cytoplasm, and thus have non-histone deacetylation activity [20]. They are divided into two subclasses: IIa (HDAC4, 5, 7, 9) and IIb (HDAC6, 10) [20]. Class III HDACs include SIRT deacetylases 1–7 and belong to a distinct family of enzymes that are not targeted by the HDACi discussed in this review. Class IV HDACs are comprised solely of HDAC11. Class I, II, and IV histone deacetylases, henceforth referred to as HDACs, rely on a zinc ion to bind their substrate and catalyze deacetylation (Figure 2). Importantly, HDACs deacetylate several other non-histone proteins important in cancer, including p53 and c-Myc [21,22] (Figure 1).

HDAC inhibitors, such as the hydroxamic acids vorinostat (a.k.a SAHA) and trichostatin A (TSA), act by binding to the catalytic site metal Zn^2+^ ion and thus blocking substrate accessibility [23] (Figure 2). Structurally, most of the HDAC inhibitors follow a general pharmacophore which mimics the natural peptide substrates and consists of: (i) a protein-surface-interacting moiety that occupies the entrance area of the active site (cap group), (ii) a hydrophobic linker that mimics the N-alkyl side chain of lysine, and (iii) a metal binding moiety that interacts with the catalytic site Zn^2+^ ion [24,25,26,27]. Hydroxamic acids, as metal binding groups, generally lead to strong inhibition. Early structural studies in HDAC-8 revealed the mode of binding of the HDAC inhibitors [23]. As shown in Figure 2, the hydroxylamates are complexed by the Zn^2+^ ion, and the aliphatic chains of the linker fit into the long hydrophobic tunnel of the active site. The terminal capping group interacts with the external portion of the enzyme and the solvent.

First generation HDAC inhibitors such as vorinostat and trichostatin are generally nonselective, targeting most of the metal-dependent isoforms [12,28]. The high degree of sequence similarity among the channels, active sites, and internal cavities of all Class I HDACs makes the design of isoform-specific inhibitors a very challenging task. However, information obtained by X-ray crystallographic data and enzymatic activity assays led to remarkable progress in the field of isoform-selective compounds for both Class I/II HDACs over recent years [25,29]. As a result, there are significant variations among HDACs’ selectivity (Table 1), which seems to stem from their chemical nature. For instance, in HDAC6, the entrance area on the enzyme’s surface is larger compared to other isoforms, and therefore selectivity for this enzyme has been achieved by incorporating large cap groups and benzyl linkers instead of aliphatic chains [30]. Selectivity of compounds across HDACs has been determined primarily through cell-free enzymatic activity assays of HDAC1, HDAC2, HDAC3, and HDAC6; and only few studies have tested all enzymes [10,11,12,13,15,16,17,18]. Interestingly, HDAC11 was reported to be a target of romidepsin, largazole [11], and entinostat [16]; however, this should be confirmed by additional studies. Overall, the complexity of histone acetylation regulation, HDAC activity, and HDACi selectivity renders this field of study particularly challenging.

### 1.2. Heterogeneity and Therapy Resistance

High grade tumors are comprised of distinct subclones of cancer cells that arise due to factors such as the tumor micro-environment, stromal interactions, and genomic and epigenomic events. During cancer progression and evolution, spontaneous genetic alterations such as mutations and copy number changes that confer fitness advantages propagate and collectively generate a heterogeneous tumor [31,32]. In addition, a wide range of epigenetic mechanisms, including DNA methylation, chromatin remodeling, and histone modifications, contribute to gene expression diversity and transcriptomic heterogeneity within tumors [33,34].

Tumor heterogeneity is recognized as a major contributor to therapy resistance [35]. Survival and adaptation of cancer cells to therapeutics relies on rewiring of biological processes. Therefore, in heterogeneous tumors, the emergence of resistant subclones is more likely than in homogeneous ones. This is supported by the fact that intra-tumoral heterogeneity stemming from mutations and copy number alterations is positively correlated with chemotherapy resistance [35,36].

Chemotherapy has remained a primary treatment for a wide variety of cancers for many decades; however, intrinsic (present at baseline) or acquired (developed after initial treatment) resistance during treatment cycles and high toxicity are major obstacles that severely limit patients’ clinical benefits [37]. Chemotherapy drugs act mainly by inhibiting processes required for cell proliferation, such as DNA replication, genome integrity, and mitosis [38]. Examples of chemotherapy include anti-folates, such as methotrexate, which causes thymidine nucleotide depletion and DNA synthesis inhibition; nucleoside analogues such as gemcitabine, which inhibits DNA synthesis and repair machinery; vinca alkaloids, which inhibit microtubule polymerization and cell division; taxanes such as paclitaxel, an antimitotic compound that promotes microtubule assembly; camptothecins and anthracyclines, which inhibit topoisomerases I and II, respectively; and platinum compounds such as cisplatin and carboplatin, which are DNA-crosslinking agents that form inter-strand crosslinks (ICLs) which induce DNA damage and inhibit DNA replication [38,39]. Radiotherapy in genotoxic cancer therapy causes a broad spectrum of DNA damage types, including double-strand breaks (DSBs), single-strand breaks (SSBs), and oxidized nucleotide adducts such as 8-oxoguanine via the formation of reactive oxygen species (ROS) [40].

Generally, cancer cells become resistant to chemotherapy through alterations in drug transport and metabolism, modulation or mutation of drug targets, and genetic rewiring to bypass or compensate for the targeted pathways [41,42,43]. For example, a well characterized mechanism of methotrexate resistance is caused by a defect in the reduced folate carrier (RFC/SLC19A1) which leads to reduced methotrexate uptake [44,45]. In the case of 5-fluorouracil, resistance has been shown to occur by overexpression of its target, thymidylate synthase [46,47]. For DNA-damaging agents such as cisplatin, increases in the cells’ capacity to repair DNA lesions, such as overexpression of genes involved in the nucleotide excision repair (NER) pathway, have been strongly linked with chemoresistance [48,49].

Analogous resistance mechanisms have been described for targeted therapeutic approaches as well. In endocrine therapy, a widely used treatment for hormone receptor-positive (HR+) breast cancer [50], several mechanisms of resistance have been identified, including loss of estrogen receptor α (ERα) expression, mutations and post-translational modifications of ERα, deregulation of ERα co-activators, and mutations in cancer-associated pathways, such as the cell cycle, tyrosine kinase signaling, and apoptosis [51]. As a result, subclones with advantageous alterations, such as defects in cell cycle checkpoints [52], can cause recurrence. Analogous resistance mechanisms have been described in response to hormone therapy of prostate cancer with anti-androgen inhibitors [53,54]. Patients carrying epidermal growth factor receptor (EGFR) mutations often develop resistance to EGFR tyrosine kinase inhibitors, through selection of subclones with mutations that activate signaling pathways that are parallel to or downstream from EGFR, such as secondary mutations in EGFR or amplification of the MET receptor tyrosine kinase locus [55,56]. In BRCA-mutant breast and ovarian cancer patients, PARP inhibitors are utilized to exploit the inherent deficiency of these tumors in homologous recombination repair [57]. In this targeted therapy, resistance or recurrence can result from subsets of cells with restored BRCA functionality, or with rewiring of DNA repair machinery to restore homologous recombination repair (HRR) function or replication fork protection [58].

### 1.3. Epigenetic Drugs to Counter Tumor Heterogeneity and Overcome Resistance

Epigenetic deregulation is a hallmark of cancer and has a major contribution to disease development and progression [33]. Transcriptional heterogeneity within tumors has often been correlated with the identification of gene expression profiles and epigenomic signatures that are linked to de-differentiation processes or to poor differentiation states that are typical among embryonic and cancer stem cell populations [59,60,61,62]. These findings suggest that the epigenetic landscape in cancer cells may drive reprogramming and compromise cellular differentiation processes. Therefore, loss of cellular identity by epigenetic deregulation in tumor subclones and cancer stem cell populations (CSCs) within tumors contributes to transcriptional heterogeneity. For instance, single-cell RNA-seq studies in glioblastoma and oligodendroglioma have demonstrated that brain tumors contain subpopulations of undifferentiated cells with stem cell signatures and subsets of cells that have undergone neural differentiation [63,64]. Since the epigenome is important for stem cell status, epigenetic dysregulation during the initial steps of carcinogenesis may also be responsible for the emergence of CSCs from tissue stem cells.

Recent studies provide a direct link between altered epigenome and drug resistance. For instance, KDM5A/JARID1A epigenetically-driven resistance to EGFR tyrosine kinase inhibitors has been reported for non-small cell lung cancer (NSCLC) [65]. Similarly, an adaptive chromatin remodeling mechanism based on the activity of KDM5A/JARID1A has been described to drive resistance to kinase inhibitors in glioblastoma CSCs [66].

Reversibility is a key feature of epigenetic modifications, and therefore targeting the dependence of CSCs and other tumor cells on specific epigenetic factors offers a therapeutic opportunity. In this direction, numerous studies have shown that pharmacological inhibition of epigenetic factors and other chromatin remodeler proteins can inhibit the expression of oncoproteins that maintain the CSC identities or the altered transcriptomes of specific tumor subclones and promote their effective elimination [67,68,69]. In addition, single-cell transcriptomic and epigenomic analyses have shown that tumor cells undergo heterogeneous adaptive responses to chemotherapy, giving rise to independent mechanisms of resistance [70]. Taken together, these findings indicate that epigenetic regulation is a core aspect of resistance emergence. As a result, targeting the epigenome could be a therapeutic strategy to mitigate mechanisms of resistance. Along these lines, Hinohara and colleagues showed that inhibition of KDM5 could mitigate anti-estrogen resistance by inhibiting transcriptional heterogeneity [71]. In addition, several lines of research suggest that epigenetic drugs can re-sensitize tumors to chemotherapy and other types of treatment (reviewed here [72,73]).

## 2. Effects of HDAC Inhibitors and Therapeutic Implications for Tumor Heterogeneity

**Figure 3:**
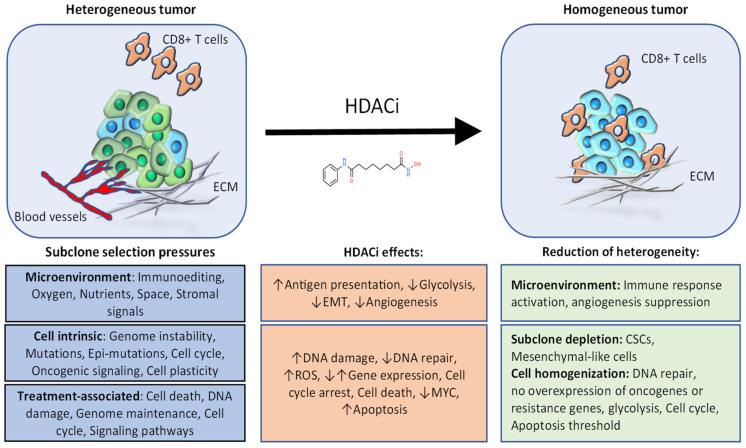
A schematic illustrating how HDAC inhibitors could be utilized to reduce heterogeneity in cancer. During cancer progression, subclone selection pressures from the microenvironment, intrinsic cellular properties and selection pressures during treatment generate tumor heterogeneity. The effects of HDACi can be utilized rationally to counter these sources of heterogeneity. Through this approach, the tumor becomes more homogeneous in certain aspects of tumor cell biology and the microenvironment, and thus responds better to cytotoxic and/or targeted therapy. EMT: Epithelial-Mesenchymal Transition; ROS: Reactive Oxygen Species; CSCs: Cancer Stem Cells; ECM: Extracellular Matrix.

Extensive research on HDACi has found that they impact most cancer-related pathways. To date, HDACi have been implicated in the regulation of numerous cellular processes, including chromatin regulation, gene expression, apoptosis, cell cycle progression, genome maintenance, DNA repair, metabolism, phenotypic plasticity, and aspects of the tumor micro-environment. This is due to the broad and complex functions of the HDAC enzymes. Through deacetylation of histones and proteins, these enzymes regulate gene expression, chromatin structure, genome replication and maintenance, and several other cellular pathways. Understanding how HDACi mediate each of their effects will be important for clinical application of these inhibitors. Rational use of HDAC inhibitors, such as in conjunction with other treatments or in selected patients, could be leveraged to reduce tumor heterogeneity and thus mitigate tumor resistance and recurrence mechanisms (Figure 3, Table 2).

**Chromatin–Gene Expression**. Histone acetylation is an integral part of chromatin regulation and is generally associated with accessible and transcriptionally active chromatin (Figure 1). Although inhibition of HDAC activity leads to increases in the global levels of histone acetylation, it does not lead to corresponding increases in gene transcription or chromatin accessibility. This is likely because the genomic distribution and recruitment of transcription factors and chromatin remodelers do not follow the same pattern.

Upon HDAC inhibition, histone acetylation does not increase uniformly. For example, in colon cancer cells, TSA and sodium butyrate cause a global increase in histone acetylation but loss of H3ac and H4ac at transcription start sites [76]. In a study where the authors evaluated the genomic distribution of H4 acetylation after vorinostat treatment, they observed a preferential increase in gene bodies [77]. Notably, this could be due to the preferential localization of the chemical probe, which has been shown to happen in the case of vorinostat [78]. In addition, several studies have noted that the pre-existing state of chromatin influences local response of chromatin to HDACi [77,79].

In the context of cancer therapy, several studies have investigated whether disrupting regulation of chromatin by HDAC inhibition has tumor-killing effects. In a model of melanoma progression, advanced melanoma displayed loss of histone acetylation in specific loci, such as cancer-associated gene promoters, and vorinostat and entinostat were found to reverse deacetylated sites [80]. HDAC inhibition was more cytotoxic in advanced melanoma, and in melanoma cell lines with reduced H3K27ac at these loci. Notably, it was not determined whether this was due to restoration of gene expression. Early studies on gene expression identified induction of apoptosis-related genes. In a CTCL clinical trial, panobinostat induced histone acetylation and the expression of p21 and other apoptosis-related genes, and downregulated proliferation-associated genes as early as 4 h after treatment [81]. Vorinostat and romidepsin were found to induce pro-apoptotic gene expression within 10 hours in a similar manner [82]. These transcriptional changes could potentially induce cell death, but it is still undetermined whether they are sufficient, and whether they are primary effects of HDAC inhibition.

An emerging concept of chromatin dysregulation in cancer is the sustenance of oncogene transcription by super-enhancers [83]. Super-enhancers, which are large clusters of transcriptional enhancers, are exploited by cancer cells to sustain oncogenic pathway activation, such as MYC. As a result, cancer cells are sensitive to disruption of super-enhancers by epigenetic drugs such as bromodomain (BET) inhibitors. Recently, several studies have reported super-enhancer disruption by HDAC inhibition. Using a systematic chemical screen, Gryder et al. identified HDAC1/2/3 as essential for core regulatory transcription, which is governed by super-enhancer activity [84]. In glioma, several HDAC inhibitors were found to reduce H3K27ac in super-enhancers of oncogenes such as MYC and PIK3C2B [85,86]. One study suggests that this is due to the redistribution of BRD4, an acetylation-binding protein that mediates super-enhancer assembly [87], to gene bodies [77]. In line with this, Kim and colleagues used global run-on sequencing (GRO-seq), a method of high-throughput analysis of nascent transcription, and showed that HDACi represses transcription elongation, thereby selectively inhibiting transcription of highly-expressed genes [88]. In contrast, another study on immediate transcriptional effects found positive effect of TSA on elongation after 10 minutes of treatment [89]. In pancreatic adenocarcinoma, HDACi inhibited TGFβ-regulated gene expression through deactivation of enhancers, and induced expression of MYC and BRD4-bound genes [90]. Overall, although there is conflicting evidence regarding how HDACi affect chromatin and gene expression, it is evident that this is caused at least partly through deregulation of enhancers.

*Therapeutic Implications*: Transcriptional dysregulation is often a driver of tumor progression, and its disruption can have therapeutic benefit. Thus, HDAC inhibitors could potentially be used to target tumors that rely on the expression of oncogenes such as c-Myc and EGFR. Additionally, transcriptional dysregulation stemming from sub-clonal events, such as genomic rearrangements (e.g., amplifications) or stochastic epigenetic events (e.g., loss of repressive state), is a significant source of intra-tumoral heterogeneity. Markedly, transcriptional heterogeneity has been associated with therapy resistance in breast cancer [71]; and in EGFR mutant NSCLC, MET overexpression is associated with resistance to tyrosine kinase inhibitors(TKI) [91,92]. Thus, HDAC inhibitors have the potential to counter heterogeneity in transcription and resistance by disrupting clonal gene overexpression—such as that of the c-Myc oncogene [86]—and the transcription of genes induced by regional signals in the tumor microenvironment, such as TGFβ [90,93]. Notably, a clinical trial in NSCLC patients examining the combination of entinostat with the EGFR inhibitor erlotinib in comparison to erlotinib alone found a small increase in progression-free survival (PFS) [94]. Currently, another trial is exploring the efficacy of the HDAC and EGFR inhibitor combination in treating TKI-resistant lung cancer (NCT02151721).

**Genome maintenance—DNA repair**. Genome stability and repair rely on strict regulation of chromatin remodeling, accessibility, and histone and DNA modifications, including histone acetylation [95]. HDACs have been shown to be indispensable for genome maintenance and DNA repair. Bhaskara et al. showed that HDAC3 is important for DNA replication, maintenance of chromatin structure, genome stability, and DNA repair by non-homologous end joining (NHEJ) and homologous recombination repair (HRR) [96,97]. These functions were attributed to modulations of global levels of histone modifications, such as H3 and H4 acetylation and H3K9me3, which are important for DNA repair, DNA replication, and heterochromatin maintenance, respectively. In addition, HDAC3 was found to be important for sister chromatid cohesion through regulation of H3K4ac [98]. HDAC1 and 2 have also been shown to be necessary for DNA replication and NHEJ [99,100]. Class II HDACs such as HDAC4 have also been implicated in DNA repair [101].

**Figure 4:**
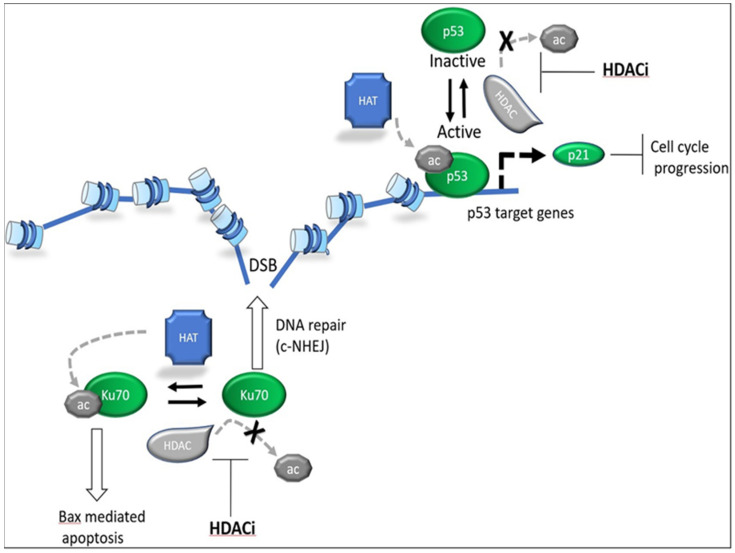
Deacetylation of p53 (e.g., at K373/K382) and Ku70 by histone deacetylases (HDACs) regulates tumor cell proliferation and DNA damage repair. DSB: double-strand break; HAT: histone acetyltransferase; c-NHEJ: canonical non-homologous end-joining.

As expected, HDAC inhibitors have been found to disrupt both genome stability and DNA repair, and several underlying mechanisms have been proposed. One study reported that vorinostat induced DNA damage through stalling of replication forks, and HDAC3 knock-down had similar effects [102]. This was attributed to aberrant replication origin firing through opening of chromatin by HDACi. Moreover, HDACi were shown to radiosensitize cells by downregulating DNA repair genes [103]. Several studies have reported that inhibition of HDACs leads to downregulation HRR components, potentially through decreased E2F1 recruitment [104,105]. In addition, HDACi were shown to modulate DNA repair by direct deacetylation of the DNA repair proteins Ku70 and PARP1 [106] (Figure 4). In leukemic cells, HDACi induced H2A.X phosphorylation 3 min after treatment, indicating the presence of DNA double-strand breaks [107]. A few studies have attributed this to the generation of ROS [108,109]. Entinostat induced ROS through loss of mitochondrial membrane potential at high concentrations as early as 2 hours after treatment [110]. In summary, it is evident that HDAC inhibitors disrupt genome stability, but the contributions of the various implicated mechanisms are unclear.

*Therapeutic Implications*. Modulation of DNA repair by HDAC inhibitors has generated great interest in cancer treatment. Several studies have shown that HDACi sensitize cells to irradiation and DNA-damaging agents [103,104]. It has been suggested that this is due to the modulation of DNA repair proteins involved in ICL repair, such as downregulation of BRCA1, Rad51, and FANCD2 [111]. Therefore, HDACi could suppress the emergence of subclones proficient at DNA repair during chemotherapy and mitigate resistance. For instance, in a heterogeneous BRCA-mutant tumor, resistance can emerge via reversion to DNA-repair defects [112]. Several clinical trials are currently evaluating this combinatorial approach, for example, in patients with advanced lymphoma (NCT01796002), glioma (NCT00268385), and solid tumors (NCT00246103).

In addition, impairment of HRR by HDACi has been found to confer sensitivity to PARP inhibition, a known characteristic of HRR-deficient cells [113]. This approach also counters the inherent heterogeneity of tumor cells in their ability to employ HRR and should therefore lead to better and sustained responses to PARP inhibitors. This approach is currently under evaluation in the clinic for ovarian, primary peritoneal, fallopian tube, and breast cancers (NCT03924245, NCT03742245). Notably, suppression of DNA repair can be detrimental for genomically unstable cells, which rely on it for repair of endogenous DNA damage. Therefore, the contributions of subclones with genomic instability to tumor heterogeneity could be eradicated by utilizing HDAC inhibitors.

**Cell cycle**. A major effect of HDAC inhibitors in cells is activation of tumor suppressor pathways and blocking of cell cycle progression. Cell cycle progression is tightly regulated to ensure homeostasis, and its obstruction can lead to senescence, apoptosis, and other forms of cell death. The activities of major tumor suppressors such as p53 and p21 are tightly linked to cell cycle regulation [114].

A number of studies have shown that HDAC inhibition induces p21 expression and cell cycle arrest [115,116]. This could be due to HDAC1 and HDAC2 inhibition, both of which have been shown to bind and repress p21 transcription [117,118], although it is not clear whether this regulation is direct. Inhibition of p53 deacetylation by HDACi has also been implicated as a potential mechanism [119]. Therefore, it has been suggested that the cell-killing property of HDAC inhibitors is mediated by transcriptional de-repression of the p21 locus. However, this hypothesis is in stark contrast to two other studies where p21 was found to confer a cytoprotective rather than cytotoxic effect in cells treated with HDAC inhibitors [110,120].

Further studies on the effects of HDAC inhibitors on the cell cycle indicated that they induce activation of the G2/M checkpoint, which has been suggested to explain why—similarly to chemotherapy—tumor cells are more sensitive to these agents in comparison to normal tissues [121,122]. HDAC3 directly regulates cell cycle progression and cyclin A degradation by modulating cyclin A acetylation [123], which points to a direct mechanism behind this effect. Overall, it is evident that HDAC inhibitors impede cell cycle progression through direct and/or indirect means, but a clear mechanistic link showing which aspects of HDAC inhibition are necessary and sufficient to block the cell cycle and induce cell death has yet to be uncovered.

*Therapeutic Implications*: An important aspect of tumor chemoresistance through heterogeneity is cell division. For example, it has been well characterized that more slowly proliferating tumor subpopulations are more resistant to chemotherapy [124]. Several studies and clinical trials have assessed combinations of HDACi with chemotherapies, such as topoisomerase inhibitors [125,126,127,128] and platinum-based compounds [108,129,130], and reported synergistic effects. This suggests that either the effect of HDACi on the cell cycle is not sufficient to promote chemoresistance or that the beneficial effects of HDACi, such as suppression of DNA repair, outweigh this drawback. In addition, HDACi could be a way to reduce cell cycle-heterogeneity in tumors and achieve robust responses to treatment. Computational models suggest that when cell cycle-heterogeneous tumors are subjected to high levels of cell death during mitosis, such as during chemotherapy treatment, slow-cycling resistant subpopulations become more prevalent [131,132]. Therefore, HDACi could suppress this selection process and block the emergence of resistant subpopulations. Further research is needed to evaluate these strategies.

Cell cycle deregulation has been identified as a resistance mechanism in endocrine therapy of ER+ breast cancer [51]. Interestingly, preclinical studies in ER+ breast cancer have associated HDAC inhibitors with suppression of ER signaling, including cyclin D signaling [133], and re-sensitization of endocrine therapy-resistant cells [134]. The combination of HDACi and the aromatase inhibitor exemestane in ER+ breast cancer patients with resistance to prior endocrine therapy provided promising results in phase II clinical trials and is currently being evaluated in two phase III trials [8,9].

**Cell Death**. Studies on HDAC inhibitor-mediated cell death showed consistent induction of intrinsic and/or extrinsic pathways of apoptosis. This type of cell death is induced by extracellular signals such as tumor necrosis factor α (TNFα) or endogenous signals such as DNA damage. Signaling cascades lead to the activation of members of the caspase family, which then target numerous proteins for proteolysis [135]. The characteristics and determinants of HDACi-mediated apoptosis that were studied varied between reports. Specifically, HDAC inhibitors have been shown to induce caspase-mediated apoptosis [136]. Meanwhile, several other studies have provided a mechanistic link between HDACi driven apoptosis and the generation of reactive oxygen species (ROS) through mitochondrial membrane depolarization in a caspase-independent manner [108,110,137]. Importantly, the latter studies showed that ROS scavengers reduced HDACi-mediated cell death. In contrast, lower concentrations of the same HDACi induced moderate cell death, but did not lead to an increase in ROS [138]. Therefore, ROS generation contributes to but is not the sole cause of cell-death by HDACi. Both vorinostat and romidepsin were found to induce expression of pro-apoptotic genes [139], and trichostatin A (TSA) treatment was shown to downregulate anti-apoptotic proteins [140]. However, it is unclear whether this effect on gene expression is direct.

Several studies have implicated p53 as a mediator of HDACi-induced cell death [21,141]. p53 transcriptional activity was shown to be negatively regulated by acetylation, and HDAC1 has been reported to deacetylate p53. Additionally, HDAC6 was shown to deacetylate p53 in hepatocellular carcinoma [142]. Moreover, downregulation of p53 has been reported by several studies [143,144]. As a result, HDAC inhibition is suggested to mediate cell death by induction of pro-apoptotic genes through p53 activation. Later studies, however, revealed that HDAC inhibitors have p53-independent cytotoxic activity. Specifically, the HDAC inhibitors vorinostat and entinostat displayed equal and partial cytotoxic activity, respectively, in p53-deficient colon cancer cells [145]. This suggests that inhibition of p53 deacetylation is not required for induction of HDACi-mediated apoptosis.

Studies have reported induction of additional types of cell death by HDACi. Vorinostat and butyrate induce apoptotic and autophagic cell death [146]. In contrast, another report noted a reduction in autophagy upon HDAC inhibition [147]. Notably, vorinostat has also been shown to induce necroptosis, a caspase-independent form of cell death [148]. Additionally, it was also suggested that HDACi cell death might be mediated by hyperacetylation of the NHEJ repair component Ku70 [149,150,151]. Ku70 acetylation and its interactions with Bax have been shown to be part of the apoptotic pathway, in a DNA repair-independent manner [152]. Although this hypothesis is appealing, it is not clear whether Ku70 acetylation is a direct effect of HDACi, nor whether it is necessary and sufficient to induce cell death. In conclusion, HDAC inhibitors have been found to induce cell death through several pathways, which could be due to direct roles of HDACs in cell death pathways or results of other effects of HDACi, such as DNA damage or gene expression dysregulation.

*Therapeutic Implications*: HDAC inhibitors consistently induce cancer cell death in vitro, which prompted their evaluation as single-agent therapies in pre-clinical and clinical settings. Despite initial success in T-cell lymphoma and multiple myeloma, most clinical trials for several solid cancers, including ovarian, NSCLC, colorectal and prostate, indicated that HDAC inhibitor monotherapy is not an effective treatment [153,154,155,156]. This could be due to poor pharmacokinetics and high toxicity profiles [157], or due to distinct genetic or epigenetic alterations present in hematological cancers [79,158]. Thus, future studies in solid tumors should focus on using HDACi in stratified settings, combinatorial approaches, or enhanced drug delivery methods.

In line with this, several groups have investigated how the modulation of p53 can enhance chemotherapy. Vorinostat has been reported to downregulate thymidilate synthase and p53 expression, thereby enhancing 5-fluorouracil chemotherapy [159]. VPA was shown to synergize with fluoropyrimidine-based chemo-radiotherapy in p53 wild-type and mutant colorectal cancer (CRC) cells [160]. These and several other preliminary studies have led to assessments of HDACi-chemotherapy combinations in the clinic (Table 3).

Resistance to therapy can emerge due to heterogeneity in the cell death threshold and kinetics [161,162]. For example, an in-silico study suggested that cell death induced by the tumor necrosis factor-related apoptosis inducing ligand (TRAIL) directly correlates with a threshold of caspase-8 activity, and that expression of anti-apoptotic proteins modulates this threshold [163]. Therefore, HDACi can be employed to lower the apoptotic threshold of subpopulations with high expression levels of anti-apoptotic genes.

**Metabolism**. HDAC inhibitors have been shown to have strong effects on cell metabolism. Several studies have reported reductions in glycolysis upon HDAC inhibition, and in one study the pentose phosphate pathway was shown to be reduced as well [86,164,165,166]. Importantly, this effect was observed by several HDAC inhibitors in several types of cancer. It is possible that it is driven by inhibition of HDAC3, which represents a major regulator of glucose metabolism and fatty acid oxidation in muscle and adipose tissue [167,168]. In another study, the authors identified a synergistic interaction between HDAC inhibition and inhibition of glycolysis [169,170], although the reason for this is not known. The link between HDAC inhibition and perturbation of metabolism could be related to the regulation of enhancers. In glioblastoma, inhibition of glycolysis by HDACi was attributed to MYC super-enhancer disruption and glycolysis gene downregulation [86]. Notably, in a proteomics approach using a vorinostat affinity probe, vorinostat was found to directly interact with enolase 1 (ENO1) and could therefore potentially inhibit its enzymatic role in glycolysis [171].

*Therapeutic Implications*: Metabolic reprogramming is known to introduce metabolic liabilities in cancer cells [172]. Therefore, dysregulation of metabolism by HDAC inhibition presents an opportunity for design of combinatorial or targeted therapeutic strategies to achieve enhanced responses. Notably, several studies have identified synergistic interactions between HDACi and inhibitors of metabolic pathways such as glycolysis, fatty acid β-oxidation, and oxidative phosphorylation in glioma [86,169]. It would be interesting to see whether tumors with inherent defects in these pathways due to specific mutations or limited nutrient availability would be more sensitive to HDAC inhibition.

Cell metabolism responds dynamically to intracellular and extracellular cues present in tumor cells, such as nutrient availability, signaling pathway activation, and gene expression. These stimuli constitute a considerable source of tumor heterogeneity that affects responses [173]. Therefore, HDACi could be applied to counter this heterogeneity, by forcing cells to conform to a certain metabolic phenotype. For example, glucose levels generate heterogeneity in cell glycolysis [174], which could be countered by using HDACi to inhibit glycolysis in all cells. This could be a way to introduce a bottleneck that limits cancer cell adaptability and resistance. In addition, induction of glycolysis after chemotherapy has been found to support cell survival and resistance in ovarian cancer [74]. As a result, HDACi-mediated inhibition of glycolysis could be leveraged to inhibit this resistance mechanism.

**Tumor Microenvironment**. The tumor microenvironment (TME) is central to cancer development, progression, and treatment [175]. HDAC inhibitors have been found to regulate several aspects of the TME that are important for clinical applications, including the stroma, innate immunity, and angiogenesis.

The advancements in immunotherapy in recent years have highlighted the importance of the tumor’s immune system components in cancer therapy [176]. HDAC inhibitors have been found to induce pleiotropic effects in the immune response, but in general they seem to promote its activation in cancer settings [177,178]. Specifically, HDACi have been shown to promote antigen presentation through induction of major histocompatibility complex (MHC) gene expression [179,180]. In addition, they have been found to modulate the expression levels of immune response-related genes in cancer cells and macrophages [181]. HDACi have been shown to promote NFκB signaling by inhibiting the ability of HDAC1 and HDAC2 to repress p65 target gene expression, and by inhibiting p65 deacetylation by HDAC3 [182,183]. Moreover, HDACs and HDACi have been shown to modulate immune cell populations and functions, such as in innate immune control of T helper 1 cells and CD4+ T cell activation [184,185]. Interestingly, a recent study reported that low-dose TSA potentiates the anti-tumor activities of tumor-associated macrophages and immune cell infiltration [186]. Overall, these effects are suggested to stem from the inhibition of HDAC activities, such as modulating gene transcription and transcription factor deacetylation.

Cancer-associated fibroblasts (CAFs), constitute another component of the tumoral stroma that have important roles in cancer treatment [187]. They induce several tumor-promoting effects, including metastasis, immune suppression, and chemoresistance [188]. HDACi have been reported to both suppress [189] and induce [190] CAF activity, which suggests context-dependent function. Further research is needed to draw conclusions.

Angiogenesis is a major aspect of tumor progression and treatment. Generation of blood vessels supports tumor growth by delivering nutrients and oxygen, and regulates other aspects of progression, such as metabolism and metastasis [191]. Several anti-angiogenesis agents are currently used for cancer treatment, and act by targeting vascular endothelial growth factor A (VEGFA) and its receptors [192,193]. HDACi have displayed anti-angiogenic effects in vitro and in vivo [194,195,196,197]. This function is attributed to several mechanisms, including transcriptional regulation of angiogenesis effectors such as VHL, HIF1α, and VEGFA; and direct interactions of HDAC1, HDAC3, HDAC4, and HDAC6 with HIF1α [198,199].

*Therapeutic Implications*: Tumor and host immune responses are central to cancer therapy and often dictate treatment responses. In the context of immunotherapy, the degree of tumor infiltration by immune cells is recognized as an important predictive factor and can be crucial for its success [200]. In addition, the composition of the immune microenvironment within the tumor can also affect the efficiency of immunotherapy [75]. Therefore, modulation of innate and adaptive immunity by HDACi presents a therapeutic opportunity. Increased tumor cell immunogenicity can boost the effectiveness of immune cells and reduce microenvironment heterogeneity by increasing immune cell recruitment in less infiltrated parts of the tumor. Interestingly, increased immune cell infiltration has been shown to be induced by HDACi [186,201], and several studies have shown that HDACi being combined with immunotherapy improves response in some solid tumors [181,202]. Clinical trials are currently investigating this approach in non-small cell lung cancer and lymphoma patients (NCT01928576; NCT03161223).

Regarding angiogenesis, the suppressive activity of HDACi is a desirable effect in the treatment of solid tumors and has been suggested as a way to suppress tumor progression and enhance therapy [197,198]. Interestingly HDACi have been shown to reduce vascularization in CTCL [203]. In addition, HDACi are under clinical investigation in combination with anti-angiogenesis inhibitors in clear-cell renal cell carcinoma (ccRCC) [204]. ccRCC is driven by VEGF signaling activation, and anti-angiogenesis inhibitors are currently used for its treatment [205,206]. Frequently, tumors develop resistance through activation of angiogenic signaling through other means, such as HIFα activation [207,208]. Therefore, the pleiotropic inhibitory effect of HDACi on angiogenesis pathways is employed to counteract these resistance mechanisms.

**Phenotypic Plasticity**. The plasticity of tumor cells in phenotypic states, such as epithelial–mesenchymal transition (EMT) and cancer stemness, contributes significantly to tumor heterogeneity [209]. EMT refers to the reversible shift of cells from an epithelial state, characterized by strong cell-to-cell adhesion, to a mesenchymal state, where cells become more migratory and invasive [210,211]. This transition affects several cellular processes and components, including the cytoskeleton, metabolism, innate immunity, proliferation, and apoptosis. In cancer this process is usually partial and is associated with metastatic disease and chemoresistance [212]. Several groups have demonstrated that HDAC inhibitors suppress the EMT transcriptional program in several cancer types, including breast, biliary tract, bladder, and others [213,214,215,216,217]. Importantly, this suppression was evident in cell lines that were predominantly mesenchymal-like, either intrinsically or due to exogenous signals such as TGFβ. This may explain why the opposite effect has also been reported in epithelial-like cancer cell lines [218,219]. Therefore, it is likely that the influence of HDAC inhibitors in EMT is context dependent, specifically on the initial phenotypic state of the cancer cells.

It is well accepted that epigenetic heterogeneity leads to transcriptional plasticity and adaptive responses to chemoresistance in cancer. Cancer stem cells (CSCs) and poorly differentiated cancer cells represent sources of cellular heterogeneity within tumors, and there is strong clinical evidence that these subpopulations are critical to conferring drug resistance [220,221,222,223]. CSCs have the potential to self-renew with symmetric or asymmetric division and are characterized by high tumor-initiating capacity [224]. Moreover, their divisions can generate differentiated progeny and transient amplifying cells, increasing the tumor’s heterogeneity. In addition, CSCs can enter a quiescent state that protects them upon treatment, since chemotherapy is more effective against proliferating cells [225]. Targeted pharmaceutical inhibition of stem cell-related signaling pathways, such as Wnt, Notch, and Hedgehog, causes high levels of toxicity, as normal tissue homeostasis also relies on these pathways [226]. Moreover, compensatory activation of other signaling pathways often confers resistance [227].

Just as in normal cells, self-renewal of cancer stem cells or proliferation of cells with undifferentiated phenotypes is highly dependent on key transcriptional programs that are regulated by specific epigenetic patterns in their chromatin [228,229]. For instance, differential DNA methylation is associated with the expression of stem cell marker genes such as CD44, CD133, and Musashi-1 (MSI1). More specifically, hypomethylation can activate these CSC genes in aggressive tumors [230,231]. Other studies in glioblastomas (GBM) have demonstrated that chromatin in CSCs is characterized by reduced levels of the silencing histone mark, H3K27me3, and possesses a more open conformation compared to non-CSCs, which together allow genes that maintain the stem cell phenotype to be expressed [232]. The overexpression of several HDACs has been associated with cancer stem cell identity, regulation of the Sonic-Hedgehog pathway, and poor survival in GBM, NSCLC, and breast and ovarian cancers [233,234,235,236]. In addition, in acute myeloid leukemia(AML), CSCs were characterized by higher H3K4me3 levels on genes involved in stem cell identity, proliferation, and metabolic reprogramming compared to non-CSCs, indicating that differentiation processes were associated with epigenetic silencing of stem cell identity genes [237].

*Therapeutic Implications.* Phenotypic plasticity such as that in the form of EMT state contributes significantly to tumor heterogeneity [209], and is considered an important mechanism of therapy resistance because it is accompanied by anti-apoptotic signaling and drug efflux [212,238]. The EMT-suppressive effect of HDACi in mesenchymal-like cells can be employed in tumors where EMT occurs and mediates resistance, such as patient subsets in breast and pancreatic cancer [239,240]. In this setting, EMT inhibition could confer several beneficial effects, including enhancements of the effects of other therapeutics, and suppression of mesenchymal subclone emergence and metastasis.

The importance of epigenetic regulation in CSCs suggests a dependence that could be exploited for cancer treatment. Specifically, disruption of chromatin states and the expression of genes required to maintain cancer stemness could be a way to target CSC populations and reduce heterogeneity [228]. Interestingly, recent studies have demonstrated that targeting the epigenetic state of the CSC pool in tumors via HDAC inhibitors can suppress the growth of cancer stem cells without impairing the functions of normal stem cells [241]. For instance, in triple-negative breast cancer, the Class I HDAC inhibitor entinostat was reported to decrease the CSC population [242]. Similarly, HDAC inhibition has been shown to reduce the cancer stem cell burden in GBM tumors [234,243,244] and NSCLC [236].

## 3. Dissecting the Variables of HDAC Inhibition

Thus far we have explored the application of HDACi in cancer treatment to counter tumor heterogeneity and achieve greater responses by targeting specific cellular processes (Table 2). However, targeted therapeutic approaches require deep understanding of the effects and mechanisms of action of the candidate drugs. At first glance, the widespread effects of HDAC inhibitors in cell biology do not suggest a unifying mechanism of action. Several questions arise when considering how HDAC inhibitors act: Which HDACs are relevant for the effects of HDACi? Why are HDACi only effective in hematological malignancies? Which effects of HDACi are primary and which are secondary? How does HDACi dosage affect the phenotypes observed? Answering these questions is vital for comprehending why HDAC inhibition is successful or is not in cancer treatment, and for the development of effective therapeutic strategies, such as countering tumor heterogeneity.

**Target selectivity**. Firstly, we must consider whether the selectivity of HDAC inhibitors influences their biological activity. Despite their name, histone deacetylases have diverse targets, which are often not histones. Moreover, HDAC inhibitors often target more than one HDAC protein, and the target specificity varies between compounds. The drugs used in clinical and pre-clinical settings of cancer treatment vary in their target specificity (Table 1). Some are selective for Class I HDACs, such as romidepsin and entinostat, and others inhibit Classes I, II, and IV to various degree, such as vorinostat and its derivatives [157]. Despite their differences in targeting selectivity, HDAC inhibitors are used in similar clinical settings and seem to have similar phenotypic effects. In addition, they invariably inhibit HDAC family members 1, 2, and 3, which suggests that inhibition of these proteins is what mediates their anti-cancer activity.

This notion is challenged by the fact that several Class II and HDAC6-specific inhibitors have shown promise as anticancer agents as well. HDAC6 deacetylates cytoplasmic proteins such as tubulin and HSP90, and has important functions in tumorigenesis, such as modulation of protein homeostasis through regulation of HSP90 and proteasomal degradation [245,246,247], p53 apoptotic activity [248], and tyrosine kinase signaling [249]. As a result, several groups have contributed to the development of HDAC6-selective inhibitors [250,251,252]. Ricolinostat, an HDAC6-selective inhibitor, was effective in models of multiple myeloma and lymphoma in vitro and in vivo [253,254]. Several clinical trials are currently assessing its efficacy in multiple myeloma and lymphoma [255] (NCT01997840; NCT02091063). Notably, ricolinostat was recently reported to mediate cell death through off-target toxicity [256], which could be attributed to its low selectivity for HDAC6 compared to Class I HDACs [253]. Another HDAC6-specific inhibitor, citarinostat, has also displayed anti-cancer activity and is being investigated in the clinic [257] (NCT02886065).

To our knowledge, few studies have directly compared how HDACi with different selectivities affect various cellular processes. In a study of p53 and NFκB signaling, the Class I/IIa selective inhibitor VPA had different effects from the HDAC6-selective inhibitor marbostat [258]. Vorinostat and romidepsin similarly induced the expression of pro-apoptotic genes [139], and cytokine expression in CTCL [259]. Sonnemann et al. reported a partial difference between entinostat and vorinostat in p53 dependency [145]. Overall, Class I and Class I/II/IV HDACi have mostly similar effects in cells, but it is probably best that HDAC6-specific HDACi are considered distinct agents. Finally, it should be noted that several effects of HDACi have been reproduced by genetic knock-down or knock-out of Class I HDACs [84,86,104,143]. Therefore, it is likely that many or most effects of HDAC inhibitors stem from inhibition of Class I HDACs.

**Tissue specificity**. Secondly, we must consider whether the activity of HDAC inhibitors is tissue-specific. So far, HDACi are being employed in the clinic only for treatment of a few hematological cancers, which could point to a tissue-specific effect. Class I and Class II HDACs have important roles in T-cell development and differentiation, which could underlie the effectiveness of HDAC inhibitors in T-cell lymphoma [158]. In addition, HDACi have been shown to target blood cancer-specific pathways, such as BCL6 overexpression in B-cell lymphoma [260], aggresome dependency in multiple myeloma [261,262], and HDAC6 overexpression in lymphoma [250].

Therefore, it is possible that HDACi have not achieved the desired efficacy as monotherapies in solid tumors because of tissue specificity, and due to inter- and intra-tumoral heterogeneity. Nevertheless, HDAC inhibitors exhibit useful biological effects in both solid and hematological cancers, that can be employed to target specific cancer alterations and improve the efficacy of other therapies. Additionally, HDACs are found overexpressed in solid cancers as well [263], which also supports the use of HDACi in solid tumor treatment.

**Dose-Dependent Effects**. Thirdly, we need to consider how HDACi dosage influences the manifestation of their phenotypic effects. Several studies have indicated differences in concentration thresholds among effects such as DNA damage and histone hyperacetylation. ROS generation and apoptosis are generally observed at high-concentrations of HDACi [110,138]. In a study on the dose-dependent effects of the Class I HDACi largazole, cell cycle blocking was observed only with higher concentrations of the inhibitor [264]. In contrast, changes in H3K9ac and H3K27ac abundance and genomic distribution were observed even at the lowest concentrations. Moreover, there was marked difference between high and low-dose HDACi in the subsets of enhancers and transcripts that were affected. In line with this, in a study testing vorinostat dosage,, histone acetylation induction was observed at lower doses than DNA damage, assessed by γH2A.X [102].

In summary, the current evidence suggests that lower levels of HDACi are sufficient to disrupt epigenetic regulation, for example, through enhancer acetylation, but more severe phenotypic effects such as genomic instability, cell cycle blocking, and apoptosis occur only by extensive HDAC inhibition. In the clinic, HDACi are administered at sufficiently high concentrations that tumor cells should display severe phenotypic effects [157]. However, it is likely that poorly vascularized regions of solid tumors are exposed to lower concentrations of HDACi, which likely impairs their cell killing effects. This might explain their success in the treatment of hematological cancers, where drug diffusion is unobstructed. Moreover, this is an additional incentive to look for combinatorial or targeted therapeutic approaches where a low dose of HDACi is sufficient. The disruption of epigenomic and transcriptional regulation observed even at low concentrations of HDACi most likely confers vulnerabilities that could be exploited for therapeutic benefit.

**Primary and Secondary effects**. Finally, we need to identify which of the effects of HDAC inhibition are primary and which are secondary. Ideally, this could be achieved by construction of a time course for all effects observed, but only a few studies have assessed phenotypic effects earlier than 16 h after HDACi treatment. The earliest events are most likely histone acetylation and DNA damage, which were observed to increase in just 3 minutes of exposure to TSA [107]. The kinetics of DNA damage generation suggest that it is likely caused by genome instability and not by indirect means, such as downregulation of DNA repair components. Generation of ROS was shown to happen as early as 2 hours post-treatment [110], but earlier timepoints have not been assessed, and it is thus not clear whether this is the underlying cause of DNA damage. TSA primed cells for enhanced induction of NFκB signaling after 1 h of treatment [183]. Effects on transcription were found as early as 1 hour post-treatment, when vorinostat was shown to significantly reduce MYC mRNA levels and cause widespread gene expression changes [77]. Two studies reported effects of HDACi on gene expression as early as 4 h and 10 min after treatment respectively, which stemmed from modulation of transcriptional elongation [88,89]. Apoptosis is generally observed after 17 h of treatment, so it is likely a secondary event.

**Figure 5:**
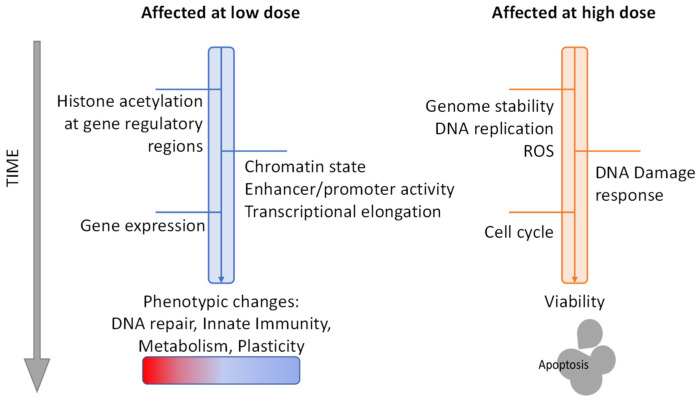
A perspective on how prominent effects of HDAC inhibitors take place in a time and dose-dependent manner based on the current literature.

Based on these findings and our knowledge so far, we can speculate on the order of events after HDAC inhibition (Figure 5). With a low dose of a HDACi, the histone acetylation increase modulates the enhancer and promoter’s chromatin structure and activity, which leads to transcriptional changes. This could explain the transcription priming observed in immune response genes. Subsequently, gene downregulation impairs metabolic and DNA repair pathways. With high doses of HDACi, additional effects are observed. Initially, strong HDAC inhibition disrupts DNA replication and/or DNA repair by histone and/or protein hyperacetylation. This leads to DNA damage, H2A.X phosphorylation, and activation of the DNA-damage response and cell cycle checkpoints. Subsequently, excessive DNA damage and an inability to resolve it, due to functional and transcriptional suppression of DNA repair, leads to apoptosis and possibly to activation of innate immunity pathways, such as antigen presentation.

## 4. Conclusions

Heterogeneity presents a major obstacle to cancer therapy. Epigenetic drugs such as HDAC inhibitors could present a way to reduce tumor heterogeneity and suppress inherent and acquired resistance mechanisms to therapeutic approaches. In this review, we examined how HDAC inhibitors affect cell processes such as chromatin regulation, gene expression, and genome stability. Significant advances have been achieved in our understanding of the underlying mechanisms, but several questions remain unanswered. In addition, we highlighted several aspects of HDACi that could be utilized for the development of new therapeutic approaches. Since their chemotherapeutic efficacy is restricted to a handful of hematopoietic cancers, we suggest that other HDACi effects, such as modulation of gene expression, DNA repair, and innate immunity, could be utilized instead.

Importantly, predictive biomarkers of gene expression, DNA repair capabilities, and the immune system’s interactions with the tumor are subject to assessment prior to treatment selection. In our perspective, significant levels of tumor heterogeneity in such biomarkers could be neutralized through precise use of HDAC inhibitors. Finally, we further considered the kinetics of HDAC inhibition, in order to deconvolute their modes of action and inform treatment regimen design. Overall, deep mechanistic understanding, a precise approach, and regimen optimization will be the key to making the most out of HDAC inhibitors as a tool for cancer treatment.

**Table 3:** Clinical trials evaluating HDAC inhibitors in cancer treatment. More clinical trials exploring drug combinations of HDACi can be found here [5,6,7]. Abbreviations: CHOP: cyclophosphamide, doxorubicin, vincristine, and prednisone; HR+: hormone receptor positive; HER2^−^: human epidermal growth factor 2 negative; CRPC: castration resistant prostate cancer; SCLC: small cell lung cancer; AML: acute myelodysplastic leukemia; MDS: myelodysplastic syndrome; DLBCL: diffuse large B-cell lymphoma; HGG: high grade glioma.

Treatment	HDACi	Drug Combination	Cancer Type	Phase	ID/Ref.
**Monotherapy**	Romidepsin		CRPC	II	[153]
			SCLC	II	[265]
	Belinostat		Ovarian cancer	II	[154]
	Vorinostat		Solid tumors	II	[155]
			Ovarian cancer	II	[156]
	Panobinostat		AML	II	NCT00880269
	Ricolinostat		Lymphoma, Lymphoid malignancies	Ib/II	NCT02091063
**Maintenance**	Panobinostat		AML, MDS	III	NCT04326764
**Chemotherapy**	Romidepsin	CHOP	PTCL	III	NCT01796002
	Vorinostat	Temozolomide	Glioma	I	NCT00268385
	Valproic acid	Epirubicin, 5-Fluorouracil, Cyclophosphamide	Solid tumors	I	NCT00246103
**PARPi**	Entinostat	Olaparib	Ovarian, Peritoneal, Fallopian tube cancer	II	NCT03924245
	Vorinostat	Olaparib	Breast Cancer	I	NCT03742245
**Endocrine therapy**	Entinostat	Exemestane	HR+ & HER2- Breast cancer	III	NCT02115282 [9,266]
	Tucidinostat		HR+ & HER2- Breast cancer	III	NCT02482753 [8]
**EGFR TKI**	Entinostat	Erlotinib	NSCLC	II	NCT00602030 [94]
	Vorinostat	Givinostat	NSCLC	I	NCT02151721
**Anti-angiogenic**	Vorinostat	Bevacizumab	ccRCC	I/II	[204]
**Multiple**	Panobinostat	Bortezomid & Dexamethasone	Multiple Myeloma	III	NCT01023308 [267]
	Entinostat	Nivolumab, Azacytidine	NSCLC	II	NCT01928576
	Romidepsin	Durvalumab, Azacytidine/Pralatrexate	Lymphoma	I/IIa	NCT03161223
	Ricolinostat	Pomalidomide, Dexamethasone	Multiple Myeloma	Ib/II	NCT01997840
	Citarinostat	PVX-410(cancer vaccine), Lenalidomide	Multiple Myeloma	I	NCT02886065
	Tucidinostat	rituximab-CHOP	MYC/BCL2 Double-Expressor DLBCL	III	NCT04231448 [268]
	Vorinostat	Bevacizumab, Temozolomide, Radiotherapy	HGG	II/III	NCT01236560

## Figures and Tables

**Table 1:** Classification, targeting selectivity (HDAC class), and applications in cancer therapy for the most-studied HDAC inhibitors.

Classification	Chemical Name	Targeted HDACs	FDA Approval
Cyclic depsipeptide	Romidepsin	Class I [10,11]	CTCL, PTCL [3,4]
	Largazole	Class I [11]	Under investigation
Hydroxamic acid	Trichostatin A(TSA)	Class I/II/IV [12,13]	Under investigation
	Vorinostat/SAHA		CTCL [1]
	Belinostat		PTCL [2]
	Panobinostat		MM [14]
Benzamide	Entinostat	Class I [12,15]	Under investigation
	Chidamide/Tucidinostat	Class I [16]	PTCL (China)
Carboxylic acid	Valproic acid	Class I/IIa [17,18]	Under investigation
	Butyric acid	Class I/II	Under investigation

CTCL: Cutaneous T-cell Lymphoma; PTCL: Peripheral T-cell Lymphoma; TSA: Trichostatin A; SAHA: Suberoylanilide Hydroxamic Acid; MM: Multiple Myeloma.

**Table 2:** Examples of how HDAC inhibitors can be utilized to mitigate specific resistance mechanisms in several types of treatment and cancer settings.

Treatment	Cancer Type	Resistance Mechanism That Can Be Suppressed by HDACi
**Chemotherapy**	Solid tumor without targeted therapy option	Clonal transcriptional heterogeneity
		Glycolysis induction [74]
**PARP inhibition**	HRR-deficient cancer	HRR activation [58]
**Checkpoint blockade inhibition**	Lung, Bladder and more	Immune surveillance evasion [75]
**Tyrosine kinase inhibitor**	EGFR+ Lung cancer	MET overexpression; EMT [55]
**Anti-estrogens**	ER+ Breast cancer	Transcriptional Heterogeneity [71]

HRR: homologous recombination repair; EGFR: epidermal growth factor receptor; EMT: epithelial–mesenchymal transition; ER: estrogen receptor.
